# AI, How Much Shall I Tell You? Exchange and Communal Consumer–AI Relationships and the Willingness to Disclose Personal Information

**DOI:** 10.3390/bs15030386

**Published:** 2025-03-19

**Authors:** Corina Pelau, Maria Barbul, Irina Bojescu, Miruna Niculescu

**Affiliations:** 1 Faculty of Business Administration, Bucharest University of Economic Studies, 010374 Bucharest, Romania; 2 Doctoral School in Business Administration I, Bucharest University of Economic Studies, 010374 Bucharest, Romania; barbulmaria15@stud.ase.ro (M.B.); bojescuelena14@stud.ase.ro (I.B.); niculescumiruna14@stud.ase.ro (M.N.)

**Keywords:** artificial intelligence, consumer, self-disclosure, communal relationship, exchange relationship, attachment

## Abstract

Personal information is an important resource for the optimal functioning of AI and technology. Starting from the different theories that define human relationships and the way information is exchanged within them, we investigate the way in which communal and exchange relationships are formed between consumers and AI and the way they influence consumers’ willingness to disclose personal information to AI. With the help of structural equation modeling, we prove empirically that attachment to AI rather develops communal relationships compared to exchange relationships between consumers and AI. Communal relationships have a stronger influence on both enjoyment and self-disclosing behavior, while exchange relationships do not trigger a self-disclosing behavior unless there is enjoyment. Furthermore, attachment to AI alone does not influence self-disclosing behavior unless a communal relationship is developed. Our structural equation model emphasized the complex nature of relationships between consumers and AI and has important implications for the way AI will be optimally integrated in business processes and society.

## 1. Introduction

The integration of AI into modern society is probably one of the most debated topics at the beginning of the 21st century (Kaplan & Haenlein, 2020; Huang & Rust, 2018). In addition to an initial fascination with the ability to create artificial intelligence (Gonzales-Jimenez, 2018), real-life experience shows that the AI integration process in society is more complex than expected (Pelau et al., 2024; Huang & Rust, 2022; Gonzales-Jimenez, 2018; Puntoni et al., 2021; Lim et al., 2023; Wu et al., 2024). Computers as Human Actors Theory (Nass et al., 1995; Reeves & Nass, 1996) suggests that individuals have the same expectations from computers or AI as they have from human beings. Based on this theory, individuals expect AI to interact and behave like human beings and several characteristics of human relationships will be transposed to human–AI interactions. As human relationships are complex, ranging from very simple transactions to complex friendship and love connections (Pelau et al., 2023), one of the main challenges of AI development is to understand the type of relationship that emerges between consumers and AI and the way it impacts their acceptance and influence on individuals.

Starting from the definition of interpersonal relations by Clark and Mills (1993), our research focuses on the way attachment to AI determines communal and exchange relationships and the way it affects interaction enjoyment and the willingness of the human consumer to disclose personal information to AI. Communal relationships occur between people who are familiar with each other and in which the parts are expected to take care of each other’s feelings and needs. Exchange relationships happen between people who are less familiar with each other and are usually more transactional, as people expect something in return for every benefit they provide to the other part (Clark & Mills, 1993). Our motivation for this topic started from the premise that depending on the attachment to AI, the consumer will develop a relationship type that will affect his/her behavior, interaction type, and self-disclosing behavior towards the AI. This is similar to human relationships, where people have higher expectations from friends with whom they are more likely to have communal relationships than from acquaintances with whom they are more likely to have exchange relationships. Based on the type of relationship, the interaction will be more or less enjoyable and the consumer will be willing to disclose more personal information or feelings (Lee & Choi, 2017; Kim & Baek, 2018; Jung et al., 2021).

## 2. Literature Review

Human relationships are complex and can be of several types. There are several classifications of human relationships, depending on the closeness and depth of the relationship. Carpenter and Greene (2015) define, for instance, four layers of relationship type based on communication. According to them, human relationships range from superficial ones to personal core relationships, having a middle and an inner relationship type in-between (Carpenter & Greene, 2015). In superficial relationships, the parties exchange general information about public topics, while middle relationships include the discussion of general social attitudes. In inner relationships, the parties start to exchange information about spiritual values and personal goals and compared to the previous relationship types, where no negative information is exchanged, they also disclose different fears and secrets. Core relationships include the exchange of personal information about feelings and private aspects of a person’s life (Carpenter & Greene, 2015). According to the same study, relationships between parties have to be reciprocal (Altman & Taylor, 1973) and can change in time (Carpenter & Greene, 2015). Another classification was performed by Reis and Patrick (1996), who divide relationships into descriptive and evaluative ones and describe the processes of developing different types of relationships.

Clark and Mills (1993) divide interpersonal relationships into two categories depending on the benefits and aid the parties receive and return in the relationship. They define communal relationships as those in which one of the parties receives a benefit without having an obligation to repay (Clark & Mills, 2012). It is characterized by the situation in which one of the parties expects the other to consider and be responsive to its needs and feelings or to be helpful and able to rely on help. These types of relationships often occur between family members or friends. Exchange relationships occur when a party feels the need to repay the other party for the received benefit. This type of relationship is more transactional and is characterized by the need to keep things “even” and by giving something in return for every benefit received (Clark & Mills, 2012). This type of relationship occurs between people who do not know each other very well, such as acquaintances or business partners.

Several theories point out the fact that information exchanged between two parties depends on the type of relationship (Altman & Taylor, 1973; Bagozzi, 1975; Pelau et al., 2024). According to social penetration theory, in closer relationships, the parties exchange more types of information and more private information (Altman & Taylor, 1973). Based on this theory, self-disclosure is a key factor in determining the type of exchanged information and it usually evolves with the development of the relationship (Altman & Taylor, 1973; Carpenter & Greene, 2015). Social exchange theory also defines the way in which information exchange is a key factor in determining human relationships (Bagozzi, 1975; Urbonavicius et al., 2021). This theory starts from the premise that exchange is one of the basic aspects of human behavior, and similar to the situation in which goods and services are exchanged, information is also an important asset that can be exchanged in a business context (Bagozzi, 1975). Especially in the context in which the optimization of different technologies such as artificial intelligence, social media, or mobile applications relies on consumer data and information, they become an important asset (Urbonavicius et al., 2021; Barth & De Jong, 2017).

There are also theories that categorize the benefits expected and received in the exchange of information. According to the social exchange theory, certain types of information are disclosed in exchange for social support, recognition, or other benefits (Bagozzi, 1975; Urbonavicius et al., 2021). These transactions can be negotiated or reciprocal. In negotiated transactions, the terms of the exchange are discussed in advance, while in reciprocal transactions a party is willing to disclose personal information expecting that the other party will have a similar behavior (Molm et al., 2000). Depending on the relationship between the parties, information exchange will take place based on trust, formal assurances, and uncertainties (Bansal et al., 2016; Mothersbaugh et al., 2012).

The idea that information can be exchanged for certain benefits is also posited by the concept of “privacy calculus”, in which consumers perform a cost–benefit analysis for sharing private information (Li et al., 2011; Dinev & Hart, 2006; Fernandes & Pereira, 2021; Wang et al., 2016). Based on this model, consumers evaluate the benefits and risks of disclosing personal information and act accordingly (Li et al., 2011; Fernandes & Pereira, 2021; Wang et al., 2016). Analyzing the factors that affect the willingness of consumers to disclose personal information, Li et al. (2011) found out that the decision is made both by affect-based and cognition-based factors, but it depends on each persons’ characteristics. In addition to material and tangible benefits obtained from providing personal data, consumers can also gain intangible benefits such as communication and social relationships (Song & Kim, 2021; Rese et al., 2020; Schroeder & Schroeder, 2018). Similar classifications are mentioned in information sharing theory, according to which the predictors for data sharing can be based on self-interest or social interaction (Constant et al., 1994), and in gratification theory, according to which consumers provide personal data for information or social purposes (Katz et al., 1973).

In spite of the great preoccupation regarding data protection and data privacy, studies show discrepancies between the attitude towards privacy protection and the actual behavior of protecting it. This discrepancy between attitude and actual behavior related to data protection is also known as the privacy paradox (Barth & De Jong, 2017; Ameen et al., 2022). Studies show that there is a great general preoccupation related to privacy concerns, but it is more difficult to relate it to a certain behavior (Li et al., 2011). Although users show great interest and worry towards privacy aspects, they do not take the necessary steps to protect their private data (Barth & De Jong, 2017). This frequently happens because consumers are not aware of the risks of providing personal data or because their benefits are higher compared to the risks (Dinev & Hart, 2006; Fernandes & Pereira, 2021; Wang et al., 2016; Hong & Thong, 2013). Situations like immediate benefits, heuristics, or social gratification can easily determine whether consumers provide personal data (Barth & De Jong, 2017; Gutierrez et al., 2023; Pallant et al., 2022). Several studies show that the desire to enhance social interactions and social relationships (Carlson et al., 2022) can also determine whether consumers provide more private information.

## 3. Hypothesis Development

Several theories emphasize the fact that consumers have similar expectations from technology as they have from human beings (Nass et al., 1995; Reeves & Nass, 1996; Altman & Taylor, 1973; Carpenter & Greene, 2015). Starting from the different theories that define human relationships, we aim to investigate how far they can be applied to consumer–AI interactions and if a similar behavior is triggered. Several studies point out the existence of parasocial consumer–AI relationships, in which AI can take different roles (Chang et al., 2023; Ki et al., 2020; Pelau et al., 2024; Moon, 2000; Youn & Jin, 2021). The role of AI as friend (Ki et al., 2020; Pelau et al., 2024), companion (Song & Kim, 2021), as well as the trust the consumer has in AI (Youn & Jin, 2021), are emphasized.

Analyzing the AI landscape, it can be observed that there are different types of robots, from personal assistants (which are owned by the consumer and are very close to him/her) (Ki et al., 2020; Hoy & Pomputius, 2018; Hsieh & Lee, 2021; Ki et al., 2020) to external service robots (which are owned by companies and are not very close to the consumer) (Wirtz et al., 2018; Ashfaq et al., 2020; Belanche et al., 2020; Zhao et al., 2025). Based on the classification of communal and exchange relationships, it can be assumed that depending on AI type, the consumer will develop different types of relationships to it (Pelau et al., 2023; Sundar et al., 2017; Liu et al., 2022). For instance, with external service robots or chatbots, the consumer is more likely to have a rather transactional exchange relationship, while with a personal intelligent assistant, the consumer is expected to have a closer, emotional, communal relationship (Chang & Kim, 2022). Similar to human relations, depending on the relationship type, the human consumer will have different expectations and different behavior towards the different AI types (Ki et al., 2020; Tsai et al., 2021).

Self-disclosing behavior is an important factor for the optimal functioning of AI and other intelligent systems (Martin et al., 2017; Song & Kim, 2021; Tran et al., 2022). An AI can personalize communication with the human consumer only if it has sufficient information from and about the consumer. For this reason, it is important to shape an AI relationship that gives the human consumer the confidence and trust to disclose personal information (Youn & Jin, 2021; Song & Kim, 2021). Studies point out the fact that the closer a relationship is, the greater the willingness of individuals to share private information (Carpenter & Greene, 2015). Based on this theory, it is expected that less information is disclosed in exchange relationships compared to communal relationships, where more personal information is disclosed (Clark & Mills, 1993; Ki et al., 2020; Tsai et al., 2021).

To better understand the formation mechanism of exchange and communal consumer–AI relationships, we have analyzed the role of attachment to AI in developing these relationship types, as well as the role of enjoyment as potential mediator in the relation to self-disclosing behavior. Based on the relationship classification by Clark and Mills (2012), communal relationships occur in the case of close family members and friends to which the individual has a higher degree of attachment. Therefore, we hypothesized that a higher attachment to AI has a stronger influence on building communal relationships compared to exchange relationships (H1).

Considering that communal relationships refer to people who are familiar with each other, in this relationship type, communication and behavior are less informal and individuals are more willing to disclose personal information (Clark & Mills, 1993). Exchange relationships occur between individuals who are less familiar with each another. In this relationship type, communication is more formal and individuals are less likely to disclose personal information (Clark & Mills, 1993). Based on this behavior, we hypothesize that communal relationships have a stronger influence on self-disclosing behavior compared to exchange relationships (H2).

In order to better understand the mechanism between consumer–AI relationships and self-disclosure, we measure the enjoyment of the interaction triggered by the two relationship types. Enjoyment is associated with a positive feeling the consumer has about the interaction and can be considered as one of the intangible benefits (Song & Kim, 2021; Rese et al., 2020; Constant et al., 1994; Katz et al., 1973) of the social relationship. Considering that a close relationship is more emotional, there is a higher probability of creating enjoyment in communal relationships compared to exchange relationships. We hypothesized that both communal (H3) and exchange relationships (H4) can be enjoyable, and, in addition, enjoyment impacts self-disclosure (H5). Moreover, enjoyment has a mediating effect on the relation between communal relationships and self-disclosure (H6) and exchange relationships and self-disclosure (H7).

**Figure 1:**
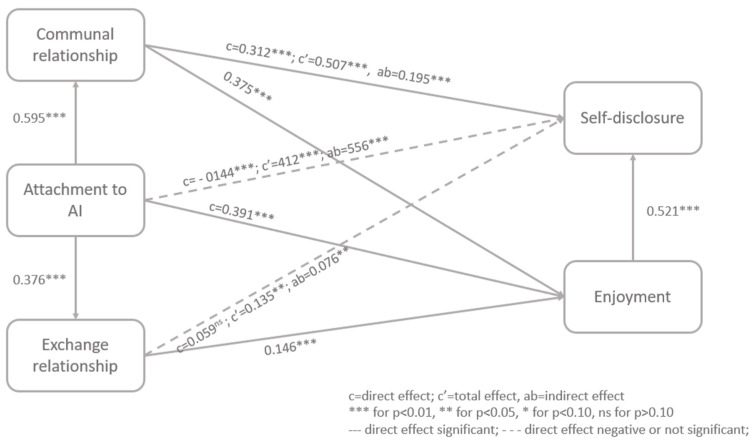
Communal and exchange relations in consumer and AI interactions.

The last analyzed aspect is if attachment is enough to determine a self-disclosing behavior or if a more complex mechanism is needed. We test empirically if attachment impacts enjoyment (H8) and self-disclosure (H9) and, moreover, if communal relationships (H10) and exchange relationships (H11) have a mediating role between attachment and self-disclosure. The empirically tested model can be observed in Figure 1.

## 4. Methodology of Research

The objective of our research is to empirically determine the way consumers’ attachment to AI forms communal or exchange relationships and further on to measure the impact of relationship type on interaction enjoyment and self-disclosure of personal information. Data collection took place with the help of an online experiment designed to measure the constructs. The respondents were recruited with the help of 12 research assistants who had to promote the link within their social group. Data collection resulted in a convenience sample of 252 valid responses. The sample included 62.7% female respondents and 37.3% male respondents. Most of the respondents were in the age group 21–40 years (78.5%), but there were also other age categories such as people between 41 and 60 years (13.8%), people younger than 20 years (5.1%), and people older than 60 years (2.3%). Taking into consideration that the age group 21–40 years is the one to be most willing to use AI, we considered the sample relevant for the research.

The respondents had to imagine that an AI they have seen in a picture has to order their coffee. The scenario of ordering a coffee was selected because it can happen in both communal and exchange relationships. One can have a friend order a coffee for them, but also someone unfamiliar can do it. In order to have different levels of attachment to AI, each of the respondents has randomly seen five types of AI with different levels of anthropomorphism and different degrees of personalization. Respondents were randomly assigned to one of the five following AI types: a service robot without any anthropomorphic features, a service robot with anthropomorphic physical features but a low degree of familiarity, a virtual intelligent home assistant without anthropomorphic features, a virtual intelligent home assistant with anthropomorphic emotional face, and an intelligent voice assistant without human physical appearance but which has a name and its own identity. For all situations, participants had to evaluate their degree of attachment, their perception of both communal and exchange relationships, as well as their enjoyment and willingness to disclose information to the AI they had seen in the picture. Attachment to AI was measured with three items adapted after Horakova et al. (2022) by including the strong bond to this AI, the great deal this AI means to the consumer, and the feeling that both AI and consumer have the same features. The items for both communal and exchange relationships were adapted after the scale developed by Clark and Mills (1993). Communal relationships were measured with seven items related to the situation in which AI considers the needs and feelings of the consumer when making decisions, as well as AI’s way of being helpful. Exchange relationships were measured with the help of three items that refer to the need to return a benefit or information after receiving, as well as keep the relationships “even”. Two items were eliminated from the original scale as they did not show sufficient loading for this item. Perceived enjoyment of the interaction with an AI was measured with five items adapted after Ashfaq et al. (2020) and Lee and Choi (2017) related to the way in which the conversation with the AI is enjoyable, funny, and exciting and the way the consumer is absorbed by the conversation and the influence it has on buying decisions. Self-disclosing behavior was measured with the help of four items adapted after Song and Kim (2021) related to the way the consumer is willing to provide personal information like name, contact and financial information, or even information about the consumer and its needs.

The processing of the data took place with structural equation modeling using the bootstrapping method with 5000 distinct samples in Smart-PLS 4.0 (Ringle et al., 2024). The reliability of the constructs is given by having higher values than the threshold for all constructs as follows: attachment to AI (Cronbach-Alpha = 0.900, CR = 0.938, AVE = 0.834), communal relationship (Cronbach-Alpha = 0.896, CR = 0.918, AVE = 0.617), exchange relationship (Cronbach-Alpha = 0.621, CR = 0.794, AVE = 0.575), perceived enjoyment (Cronbach-Alpha = 0.928, CR = 0.946, AVE = 0.779) and self-disclosing behavior (Cronbach-Alpha = 0.894, CR = 0.926, AVE = 0.759). Convergent validity is ensured by having all outer loadings higher than the threshold of 0.700, as can be observed in Table 1. Discriminant validity is confirmed by all values of the heterotrait–monotrait criterion less than 0.900.

## 5. Results

The results of the structural equation model show that consumers’ attachment to AI has a stronger influence on building communal relationships (β = 0.595, t = 13.243, *p* = 0.000) compared to exchange relationships (β = 0.376, t = 6.303, *p* = 0.000). This highlights the fact that consumers expect AI to be helpful and careful to their needs without giving anything in return. Communal relationships have a direct positive significant effect on both enjoyment (β = 0.375, t = 6.148, *p* = 0.000) and self-disclosure (β = 0.312, t = 4.025, *p* = 0.000) and enjoyment positively affects self-disclosure (β = 0.521, t = 6.489, *p* = 0.000). Furthermore, enjoyment mediates the relationship between communal relationships and self-disclosure, having a total effect of c = 0.507 (t = 7.650, *p* = 0.000) and a significant indirect effect of ab = 0.195 (t = 4.298, *p* = 0.000, CI = [0.113; 0.290]). Compared to this, exchange relationships have a lower significant influence on enjoyment (β = 0.146, t = 2.763, *p* = 0.006) and no significant direct effect on self-disclosure (β = 0.059, t = 0.916, *p* = 0.360). However, enjoyment can change this result by having a total mediating effect c = 0.135 (t = 2.069, *p* = 0.039) and a significant indirect effect ab = 0.076 (t = 2.484, *p* = 0.013, CI = [0.024; 0.144]) in the relationship between exchange relationships and self-disclosing behavior. This result shows that, indeed, attachment to AI leads to a communal relationship, which has a greater influence on both enjoyment and self-disclosure. In opposition to this, exchange relationships can lead to self-disclosure only if there is an enjoyable interaction between consumers and AI. These relationships can be observed in Table 2 and Figure 1.

Attachment to AI has a positive significant effect on enjoyment (β = 0.391, t = 6.644, *p* = 0.000) but a negative significant effect on self-disclosure (β = −0.144, t = 2.403, 0.016). This shows that having a stronger bond to AI does not necessarily imply that the consumer is willing to disclose personal information with it. On the contrary, it reduces the amount of disclosed information, as the consumer might feel exposed to judgement or manipulation. However, enjoyment and the building of a certain type of relationship can change the situation, leading to a total effect of c = 0.412 (t = 7.447, *p* = 0.000) and a cumulative significant indirect effect of ab = 0.556 (t = 11.299, *p* = 0.000, CI = [0.464; 0.654]).

There are also some specific effects that need to be mentioned. Both communal (β = 0.223, t = 4.465, *p* = 0.000) and exchange relationships (β = 0.055, t = 2.271, *p* = 0.023) have a mediating effect in the relationship between AI attachment and enjoyment. This specific effect is higher for communal relationships (β = 0.223) compared to exchange relationships (β = 0.055). For the relation AI attachment–self-disclosing behavior, there is a significant mediation effect for communal relationships (β = 0.185, t = 3.939, *p* = 0.000), but the same is not valid for exchange relationships (β = 0.022, t = 0.883, *p* = 0.377). This again shows that self-disclosing behavior is more influenced by communal relationships than by exchange relationships. These specific indirect effects can be observed in Table 3.

The relevance of the effect is also shown by the high R-squared values. Attachment to AI determines an R-squared value of 0.354 in relation to communal relationships and a value of 0.141 in relation to exchange relationships, highlighting the greater effect on communal relations. Attachment to AI and the two types of relationships determine an R-squared value of 0.598 in relation to enjoyment and all cumulated determine an R-squared value of 0.511 in relation to self-disclosing behavior. These values can be observed in Table 4. These values show the impact of attachment and relationship type on self-disclosing behavior.

## 6. Discussion

The results of our research emphasize the fact that, similarly to human interactions, consumer–AI relationships can be of different types. As our results show attachment to AI has a stronger influence on a communal relationship between consumers and AI, in which the consumer expects AI to be helpful and careful with his/her needs without giving anything in return. The influence of AI attachment is lower for exchange relationships, in which, for every received benefit, something has to be given back. This is similar to human relationships, where in family or friendship relationships mutual trust and communication are expected, while for more distant acquaintances, mainly transactional relationships take place (Clark & Mills, 1993). This result is in line with the existing literature according to which in functional consumer–AI relationships an exchange type of relationship is expected, while in social consumer–AI relationships a communal type of relationship is desired (Chang & Kim, 2022).

The type of relationship has important implications for enjoyment and self-disclosing behavior. The effect of communal relationships on enjoyment is higher compared to exchange relationships. Communal relationships trigger self-disclosing behavior compared to exchange relationships, in which there is no significant effect on self-disclosure. Both results are similar to human behavior, where individuals enjoy better the companionship of friends and family and less the ones of acquaintances or people they do not know very well. In the same way, communication with people we know well is more informal and more personal information is disclosed. This result has important implications on the ways relationships with consumers are built, as it means that in order to make the consumer disclose personal information, it is important to develop a communal relationship between him/her and AI. In both types of relationships, enjoyment mediates the relationship with self-disclosure. For exchange relationships, the total mediating effect emphasizes the importance of enjoyment as a key factor in determining whether the consumer discloses personal information. The consumer will be willing to disclose personal information only if there is a certain degree of enjoyment. For communal relationships, enjoyment increases the self-disclosure behavior of the consumer towards AI, but it has a smaller effect compared to exchange relationships because in this case mutual trust is the construct that defines the relationship and not enjoyment. In communal relationships, enjoyment is a component but not the main reason for disclosing personal information, while in exchange relationships, enjoyment can trigger positive feelings that can increase the willingness to disclose personal information.

From a practical point of view, the results have important implications on the way the different AI types are designed. Personal AI assistants or intelligent home assistants must be developed with the ability to be empathic and build communal relationships in order to make the consumer disclose personal needs and desires for personalizing the experience. External chatbots or service robots have to be developed to be enjoyable in order to make the consumer disclose personal needs and wishes for efficient service delivery. Even if they do not necessarily develop a close relationship with the consumer, by being funny, empathic, and enjoyable, they can interact and receive the necessary information to fulfill the expectations of the consumer. In addition to this, the type of communication will differ from one type of AI to another. For instance, personal AI assistants will have a more informal type of communication, being allowed to ask more personal information from the consumers, compared to external service robots, which will ask only general questions about the product or service they provide to the consumer.

Regarding the relationship between attachment to AI and self-disclosure, it can be observed that attachment alone does not influence consumer self-disclosing behavior. Although exchange relationships do not influence much the relation, communal relationships mediate the relation between attachment to AI and self-disclosure. This means that only by defining a communal relationship in which mutual benefits are expected can attachment influence self-disclosure. The negative influence of attachment on self-disclosure can be explained by the fact that people might feel exposed disclosing personal information unless there is a defined relationship of either exchange or mutual trust. Only designing a cute, emotional AI is not enough to make the consumers disclose personal information, but a clear relationship has to be defined. This highlights even more the importance of defining clear relationships between consumers and AI. The fact that attachment alone impacts negatively self-disclosure is another confirmation that a clearer relationship has to be developed in order to be willing to tell personal information. This result is also in line with previous research, according to which empathetic behavior influences self-disclosure only if there is trust or control over the disclosed information (Pelau et al., 2024). It also confirms existing theories such as the Computers as Social Actors Theory (Nass et al., 1995; Reeves & Nass, 1996), according to which people have the same expectation from technology as they have from human beings, and consequently they relate in a similar way to each other. This, again, has important implications for the way relationships between people and AI are programmed.

## 7. Conclusions

In a business context, information about consumers’ needs and desires is an efficient resource to personalize services and deliver the expected products. Self-disclosure is a key source of information in this process and the best way to optimize both product co-creation and business–consumer interactions. Considering that AI is increasingly used and implemented in several business processes, it is essential to design an optimal type of AI that has the ability to collect key information from consumers and, at the same time, deliver the expected products. For this reason, it is important to study AI-related factors that affect the willingness of consumers to interact and disclose personal information. Starting from the example of human relationships, where the close and extended social circles have an influence on buying decisions and consumer behavior, it is important to study if this situation can be transposed to AI. People have close family members and friends who recommend different products and services and, at the same time, there are more distant company representatives or service employees who can provide the right information to convince the consumer to make a buying decision. In both situations, the social circle has an influence on the consumer but in different ways. In a similar way, AI can take different roles in influencing the buying decisions of consumers. For this reason, it is important to understand the roles AI takes in the life of consumers, as well as the relationships that occur between them and optimizing this relationship. Our research results emphasize these different relationships between consumers and AI and analyze the way they influence self-disclosing behavior, having both theoretical and practical implications.

From a theoretical point of view, our research will extend several existing theories by transposing human exchange and communal relationships (Clark & Mills, 1993) to consumer–AI interactions. The main theoretical contribution lies in defining consumer–AI relationships depending on attachment, expectations from another, and exchanged information. Our research confirms that communal and exchange relationships can be transposed to consumer–AI interactions and that they have a role in the amount and content of exchanged information. Moreover, it extends the Computers as Social Actors Theory (Nass et al., 1995; Reeves & Nass, 1996) by adding relationship types to consumers’ expectations towards AI. Consumers not only have similar expectations from technology and computers, but they also develop human-like relations to their AI. This result will help develop and program AI roles that integrate them best into the consumer’s life. Understanding the relationship type between consumers and AI contributes to an efficient AI design for each type of relationship. In addition to all these, our research results also add relationship type as a factor for the willingness to disclose personal information to AI.

From a practical point of view, our research has a wide range of applications for the successful implementation of service robots and intelligent personal assistants in the business environment. First of all, it defines the relationship type and the role AI takes in the lives of consumers. Depending on this, the type and amount of information provided by the consumer is set in order to optimally design AI’s communication and behavior towards the consumer. This is important in order to balance the amount of gathered information without interfering in the private sphere of the consumer. In communal relationships, typical for social interactions performed by consumer-owned devices such as the personal virtual assistants of home assistants, a more private interaction is allowed. In exchange relationships typical for functional purposes, such as chatbots or service robots, a more distant and formal communication is expected. This result is important to program AI’s communication type and style. An AI that develops a communal relationship will be programmed to talk more and to connect better to the consumer and will be allowed to ask more private information, while an AI that develops exchange relationships will have a more topic-oriented communication, with shorter sentences and less personal information about the consumer. This communication optimization is a second practical implication that resulted from this research. A third important practical implication is the involvement of companies in designing AI algorithms that influence the consumer behavior. If AI has the ability to influence the consumer by being a friendly companion or an informed service agent, the question which arises is what a company wants to influence. Knowing the different types of consumer–AI relationships can help companies to develop the right algorithms in order to influence consumers’ buying decisions.

In this situation, there is an ethical concern regarding the existence of a close consumer–AI relationship, as consumers might be easily manipulated by a simulated para-friendship to AI. For this reason, an important topic that should be analyzed in the future is about who has control over the algorithm inside the AI. There are already different studies that point out the role that AI may take in business–consumer interactions, from simple recommendation systems to the situation in which AI takes over the buying decisions (Huang & Rust, 2022; Pelau et al., 2023). In this context, it is even more important to define consumer–AI relationships, as, depending on it, consumers will be willing to delegate some of their decisions. It can be seen that close consumer–AI relationships can have a reversed side by developing mechanisms and regulations in order to protect consumers from these interferences. The AI Act developed by the European Commission already defines the potential categories of risks to which consumers might be exposed. The risk of manipulation, deception, and exploitation of vulnerabilities are categorized as some of the most harmful AI-related risks (European Commission, 2024). For this reason, it is even more important to understand the types of relationships that might occur or be induced between consumers and AI and to increase the regulations of what is and is not allowed. It is also important to raise awareness regarding these developments, as consumers are the ones that can decide how far they are willing to develop close relationships to AI, disclose personal information to AI, and let it intervene in their buying decisions.

Emphasizing the diversity of the AI landscape is a fourth important practical implication that resulted from this research. Many existing studies focus on the general acceptance of AI, but in fact there are different types of AI, with different functions and roles, that develop different relationships to the consumer. The limitation of our study lies in the use of a convenience sample for defining the hypothesized relationships. Future studies should include more diverse demographic groups to generalize findings and elaborate even more on the types of consumer–AI relationships. Understanding these relationships will bring important insights in AI-oriented research and also in the future development and optimal implementation of AI, as well as in developing proper regulations for AI integration into society and into the business world.

## Figures and Tables

**Table 1:** Reliability of constructs and outer loadings.

Construct/Item		Outer Loading
**Attachment to AI** (adapted after Horakova et al., 2022) Cronbach-Alpha = 0.900, CR = 0.938, AVE = 0.834		
	I have a strong bond to this AI	0.908
	This AI means a great deal to me	0.934
	I feel like the AI and I have the same features	0.897
**Communal relationship** (adapted after Clark & Mills, 1993)		
Cronbach-Alpha = 0.896, CR = 0.918, AVE = 0.617		
	It bothers me when the AI neglects my needs	0.757
	When making a decision, I take the AI’s needs and feelings into account	0.743
	I believe that the AI should go out of their way to be helpful	0.745
	I expect the AI I know to be responsive to my needs and feelings	0.844
	I often go out of my way to interact with the AI	0.867
	When I have a need, I turn to AI for help	0.760
	When I have a need that the AI ignores, I am hurt	0.775
**Exchange relationship** (adapted after Clark & Mills, 1993)		
Cronbach-Alpha = 0.621, CR = 0.794, AVE = 0.575		
	When I give information to the AI, I generally expect something in return	0.512
	When people receive benefits from the AI, they ought to repay the AI right away	0.890
	It is best to make sure things are always kept ‘even’ in the relation with the AI	0.818
**Perceived enjoyment** (adapted after Ashfaq et al., 2020; Lee & Choi, 2017)		
Cronbach-Alpha = 0.928, CR = 0.946, AVE = 0.779		
	I enjoy the conversation with the AI	0.898
	It is fun and pleasent to share a conversation with the AI	0.905
	The conversation with the AI is exciting	0.926
	I was absorbed in the conversation with the AI	0.909
	I enjoy choosing products more if they are recommended by an AI than if I choose them myself	0.766
**Self-disclosing behavior** (adapted after Song & Kim, 2021)		
Cronbach-Alpha = 0.894, CR = 0.926, AVE = 0.759		
	I am willing to provide personal information such as name, e-mail address, and phone number to the AI	0.878
	I am willing to share my financial information such as debit/checking and credit card numbers with the AI	0.821
	I am willing to provide the AI with information about me	0.921
	I am willing to provide the AI with information about my product needs	0.863

**Table 2:** Path coefficients and direct, indirect, and total effects in the structural equation model.

Relation	β	t	*p*
Attachment to AI → Communal relationship	0.595	13.243	0.000
Attachment to AI → Exchange relationship	0.376	6.303	0.000
Communal relationship → Enjoyment	0.375	6.148	0.000
Communal relationship → Self-disclosure	0.312	4.025	0.000
Enjoyment → Self-disclosure	0.521	6.489	0.000
Communal relationship → Enjoyment → Self-disclosure (total effect)	0.507	7.650	0.000
Communal relationship → Enjoyment → Self-disclosure (indirect effect)	0.195	4.298	0.000
Exchange relationship → Enjoyment	0.146	2.763	0.000
Exchange relationship → Self-disclosure	0.059	0.916	0.360
Exchange relationship → Enjoyment → Self-disclosure (total effect)	0.135	2.069	0.039
Exchange relationship → Enjoyment → Self-disclosure (indirect effect)	0.076	2.484	0.013

**Table 3:** Specific indirect effects in the structural equation model.

Relation	β	t	*p*
Attachment to AI → Communal relationship → Enjoyment	0.223	4.465	0.000
Attachment to AI → Exchange relationship → Enjoyment	0.055	2.271	0.023
Attachment to AI → Communal relationship → Self-disclosing behavior	0.185	3.939	0.000
Attachment to AI → Exchange relationship →Self-disclosing behavior	0.022	0.883	0.377

**Table 4:** R-squared values for specific relationships.

Relation	R-Squared
Attachment to AI → Communal relationship	0.354
Attachment to AI → Exchange relationship	0.141
All variables → Self-disclosing behavior	0.598
All variables → Enjoyment	0.311

## Data Availability

Data can be made available upon request.
